# What Is on Your Mind? Impaired Social Cognition in Primary Central Nervous System Lymphoma Patients Despite Ongoing Complete Remission

**DOI:** 10.3390/cancers13050943

**Published:** 2021-02-24

**Authors:** Milena Pertz, Thomas Kowalski, Patrizia Thoma, Uwe Schlegel

**Affiliations:** 1 Department of Neurology, University Hospital Knappschaftskrankenhaus, Ruhr University Bochum, In der Schornau 23–25, D-44892 Bochum, Germany; thomas.kowalski@kk-bochum.de (T.K.); uwe.schlegel@kk-bochum.de (U.S.); 2 Neuropsychological Therapy Centre (NTC)/Clinical Neuropsychology, Faculty of Psychology, Ruhr University Bochum, Universitätsstraße 150, D-44780 Bochum, Germany; patrizia.thoma@rub.de

**Keywords:** primary central nervous system lymphoma, neuro-oncology, quality of life, social cognition, empathy, social problem-solving

## Abstract

**Simple Summary:**

Prolonged survival after treatment of primary central nervous system lymphoma (PCNSL) led to considering patients’ everyday functional needs. Apart from cognitive functions (e.g., memory, attention), which have been investigated previously, social participation affects the quality of life (QoL). Although successful navigation in a social world is crucial for participation, social functioning in PCNSL patients has not been addressed so far. In this study, we investigated social abilities in PCNSL patients with ongoing complete remission for at least one year. PCNSL patients had difficulties in inferring others’ mental states and were impaired in providing optimal solutions for difficult social situations as compared to matched healthy controls. This demonstrates that PCNSL patients differ from healthy controls in their social functioning even in the absence of (residual) disease itself. Social difficulties may represent an additional burden affecting patients’ and caregivers’ QoL.

**Abstract:**

Within the past decades, long-term survival was achieved in a substantial fraction of primary central nervous system lymphoma (PCNSL) patients, expanding the focus of research to their quality of life (QoL). Social relationships crucially contribute to well-being in the context of adversity. Therefore, abilities that facilitate social interactions essentially determine QoL. The present study specifically targeted those sociocognitive abilities. Forty-three PCNSL patients with ongoing complete remission to therapy for at least one year and 43 healthy controls matched for age, gender and education were examined with standardized self-report and behavioral measures of social cognition. An impaired ability to comprehend others’ feelings was found in patients for both positive and negative mental states. Patients had difficulties in identifying the awkward element in challenging social situations, whereas the degree of discomfort experienced in those situations was comparable between groups. Both the production of optimal solutions for social situations and the mere recognition of these among less optimal strategies were impaired in patients. Clinicians should be aware of possible sociocognitive impairment and ought to address this in additional supportive interventions. Impaired sociocognitive abilities may entail social conflicts at a time when patients rely on social support. This, in turn, could detrimentally affect QoL.

## 1. Introduction

Primary central nervous system lymphomas (PCNSL) represent aggressive extranodal non-Hodgkin’s lymphomas and account for approximately 3% of all primary intracranial tumors [1]. Chemotherapy protocols have improved prognosis significantly [2,3], especially in patients younger than 65 years [4,5]. Due to this, the previous focus on progression-free and overall survival has gradually expanded to a broader analysis, including quality of life (QoL) as a relevant additional endpoint to any therapy [6,7,8,9].

As an important aspect of QoL, neurocognition has been investigated intensively and appeared to be preserved in PCNSL patients treated with chemotherapy alone [8,10]. However, despite improved prognosis and reduced neurotoxicity in a substantial fraction of PCNSL patients [5,6], data on social functioning are sparse and inconsistent. Everyday human life is characterized by social interactions, whose success mostly depends on the ability to empathize with others, recognize social conflicts as such and appropriately address them. Large parts of our brains have evolved to deal with social interactions. The evolutionary history of the human brain suggests that social mechanisms are very ancient and shared with other animals [11]. The main areas referred to as the social brain encompass the medial prefrontal cortex, superior temporal sulcus, temporal-parietal junction, amygdala and insula [12]. Social relationships contribute to resilience [13]—i.e., the ability to maintain high levels of functioning in the context of adversity—and are known to buffer the adverse effects of illness on psychological well-being [14]. Social cognition is an umbrella term for mental processes associated with interpersonal interactions [15], facilitating adequate social behavior and fostering social relationships, consequently contributing to QoL [16]. Indeed, social cognition is a complex cognitive construct that allows to decode and encode the social world [17]. Sociocognitive functions promote an individual’s ability to form part of a social group. They encompass several different, yet interrelated, skills and constructs ranging from more elementary processes, such as emotion recognition, to more complex ones, such as empathy, Theory of Mind (ToM), and social problem-solving [15]. The two higher-level processes that were addressed in this paper are empathy and social problem-solving. Empathy denotes an individual’s understanding of and emotional reaction to the observed or imagined emotional experiences of another individual [18]. Empathy can be subdivided into the ability to vicariously experience and respond to another person’s emotional state [19] (emotional empathy) and to cognitively understand another person’s feelings (cognitive empathy) [20]. Cognitive empathy is frequently used synonymously with the term affective ToM [20], which describes the ability to infer and understand the emotional state of others [21]. Empathic reactions may elicit situation-specific (e.g., prosocial) behavior. Social problem-solving denotes the ability to recognize an interpersonal conflict as such, to generate possible solutions and to implement the most appropriate one [22]. Theoretical models postulate that social problem-solving involves (1) perceptual skills (e.g., emotion recognition), (2) cognitive aspects (e.g., empathy and ToM) and (3) performance-based processing skills [22]. The latter involves the generation of possible solutions to a problem and the choice of the most appropriate alternative [22,23]. Empathy and social problem-solving are interrelated: As the best solutions are usually those that solve the conflict on a practical level, but also in a socially sensitive manner, empathic understanding of mental states forms a prerequisite for effective problem-solving [22].

Impairment of sociocognitive functions can cause misunderstandings about others’ intentions and lead to inappropriate responses in social interactions. Impaired social cognition was found to negatively affect the QoL of several clinical conditions, such as post-traumatic stress disorder [24,25], schizophrenia [26,27] or autism [28,29].

Although sociocognitive impairment is linked to decreased QoL and represents a “core cognitive phenotype” of developmental, psychiatric and neurological disorders [30,31], social cognition has sparked little interest in neurooncology, e.g., [32,33,34,35,36,37,38,39] (for a review, see [40]). Previous studies mainly focused on patients undergoing surgical interventions and reported that patients with low-grade tumors were preoperatively marginally affected only, whereas patients with high-grade tumors showed sociocognitive impairment before treatment but no additional effects of resection [33,35,38]. Although the extent of postoperative impairment was described as moderate and transient in most studies, sociocognitive impairment was found immediately after resection [34,41]. The previous studies partly lacked control groups [34], assessed patients only preoperatively [39], and included small sample sizes [36] or heterogeneous patient groups [33,35,39]. Both self-report [41] and behavioral measures [34,36] were used in neuro-oncological patients but rarely in combination. The most intensively researched aspects of social cognition in brain tumor patients are emotion recognition, empathy and ToM. Data on higher sociocognitive functions in brain tumor patients, for instance, social skills and social problem-solving, are scarce. In PCNSL patients, psychosocial aspects were addressed in one early study only: Among other outcome measures influencing QoL, the Social Adjustment Scale (self-report) and the Problem-Solving Inventory have been used in 11 out of 20 eligible PCNSL patients with complete remission (CR) after initial treatment with methotrexate-based chemotherapy without radiation. The study reported comparable ratings relative to normative data [9]. However, there was no performance-based assessment. In general, there is a complete lack of data for behavioral emotional empathy and for social problem-solving in any group of brain tumor patients and in PCNSL patients in particular.

The present study was the first that aimed to investigate empathy and social problem-solving in a group of PCNSL survivors. Both behavioral and self-report measures of cognitive and emotional empathy were administered to 43 PCNSL patients with ongoing CR to chemotherapy alone and to 43 pairwise matched healthy controls. Additionally, our study provides a fine-grained analysis of how PCNSL patients interpreted and dealt with difficult interpersonal situations by focusing both on the quality of freely generated problem-solving strategies and on the ability to identify optimal solutions when presented, among others. Based on previous reports of psychological burden (i.e., depression and anxiety) [9] and of difficulties in reintegration into work and everyday life after successful therapy of PCNSL [42], one may assume that sociocognitive impairment partly accounts for those difficulties. Therefore, we hypothesized that PCNSL patients might be impaired in understanding the emotional experiences of interaction partners and in providing appropriate solutions for interpersonal problems preventing them from interacting adequately with their social environment. The free generation of appropriate solutions for social problems was assumed to be more impaired than their mere recognition.

## 2. Materials and Methods

### 2.1. Participants

Between October 2018 and March 2020, 43 PCNSL patients treated in our institution with CR to chemotherapy without whole-brain radiotherapy (WBRT) were included in this study (Table 1). Inclusion criteria were as follows: PCNSL patients were included if they were diagnosed with PCNSL and presented with ongoing CR or CR unconfirmed (CRu) for at least one year after completion of chemotherapy without WBRT. To ensure a general understanding of instructions, an estimated overall intelligence score above 80 was an inclusion criterion for all participants. None of the participants who gave written informed consent to participate presented an estimated overall intelligence score below 80. Therefore, none of the participants was excluded from the analyses. An exclusion criterion for this study represented the application of WBRT. An exclusion criterion for healthy controls was the presence (current or lifetime) of traumatic brain injuries, neurological or psychological diseases.

In all 43 patients, CR or CRu was ongoing for at least one year (median 35 months, range 12–98 months). Five patients of this series suffered from a cerebral tumor relapse and one from an ocular relapse, in all of whom salvage treatment led to CR for more than one year prior to participation. Median time after completion of first-line treatment and tumor relapse was 27 months (range 7–137 months; Text Sl). Forty-three matched healthy matched controls were recruited per advertisements placed in a regional newspaper and via addressing acquaintances explicitly searching for individuals without any severe health conditions (current or lifetime). Neither traumatic brain injuries nor neurological or psychological diseases (current or lifetime) were reported by any healthy control. The healthy volunteers were matched pairwise to the patients’ age, gender and education.

The patients (*n* = 43, 21 females) showed a median age of 65 years at time of participation (range 37–83 years). The healthy controls (*n* = 43, 21 females) showed a median age of 66 years (range 35–80 years). PCNSL patients and healthy controls were paired matched for age (*p* = 0.955), gender (*p* = 1.0), years of school (*p* = 0.064) and years of education (*p* = 0.351). A repeated-measures analysis of variances (ANOVA) was computed to analyze number of words in the Regensburg verbal fluency test, with group as between-subject and fluency condition as within-subject factor. Patients and healthy controls overall significantly differed in verbal fluency (*F*(1,84) = 6.077, *p* = 0.016, *η*^2^ = 0.067) with an overall higher number of words generated in the healthy control group (*p* = 0.016), irrespective of condition. Besides the main effect of group, a main effect of fluency condition occurred (*F*(2,168) = 229.433, *p* < 0.001, *η*^2^ = 0.732) with a lower number of words generated for phonematic fluency as compared to both conditions of semantic fluency (both *p-*values < 0.001) as well as a higher number of words generated for semantic fluency one category as compared to semantic fluency category switch (*p* < 0.001), as revealed by Bonferroni-corrected *t-*tests. The interaction was not significant (*p* = 0.214). PCNSL patients showed significantly higher Beck Depression Inventory (BDI) scores (*t*(55.5) = −3.420, *p* = 0.001, *d* = 0.738) and significantly lower overall estimated intelligence scores (*t*(82) = 2.780, *p* = 0.007, *d* = 0.607) than healthy controls (Table 2).

### 2.2. Screening Measures

All participants completed a pre-assessment questionnaire. This questionnaire assessed demographic data, medical history, and current health status with a focus on neurologic and psychiatric illnesses in the history as well as on the use of drugs acting on the nervous system. In the healthy control group, the presence of psychiatric or neurologic illnesses (current or lifetime) was also ruled out based on that questionnaire. Overall intelligence of at least 80 was estimated with a German multiple-choice vocabulary intelligence test [43] which provides a measure of premorbid verbal intelligence. This test has been previously used in PCNSL patients to estimate premorbid intelligence [6]. Also, all participants were assessed with the following neuropsychological background measures: Verbal phonematic and semantic fluency was assessed with the German Regensburg verbal fluency test [44] to control for overall verbal fluency when assessing social problem-solving fluency. Participants were asked to name as many words as possible with the initial letter “P” (phonematic verbal fluency), as many animals as possible (semantic verbal fluency one category) and generate as many words as possible alternating between sports and fruits (semantic verbal fluency category switch), each within one minute. The number of words generated according to these rules represented the dependent variable in the respective conditions. To screen for the severity of depressive symptoms, the German variant of the revised BDI [45] was used. Participants were asked to indicate their agreement or disagreement with each of 21 statements representing depressive symptoms (e.g., insomnia, listlessness, worthlessness, sadness) within the last two weeks on a bipolar four-point rating scale. Summed scores were indicative of the severity of depressive symptoms and were used as the dependent variable.

### 2.3. Social Cognition Measures

#### 2.3.1. Dispositional Empathy (Interpersonal Reactivity Index)

Dispositional empathy was assessed with the German abbreviated variant [46] of the Interpersonal Reactivity Index (IRI) [47]. The IRI [46] encompasses 16 items forming four subscales assessing distinct aspects of trait empathy by self-report: The two cognitive subscales assess the ability to mentally project oneself into fictional characters and situations (“fantasy”) and to adopt other peoples’ mental perspectives in everyday life (“perspective-taking”). The emotional empathy subscales gauge feelings of compassion and concern for unfortunate others (“empathic concern”) as well as the tendency to respond with personal emotional discomfort to other peoples’ suffering (“personal distress”) [21]. Participants indicated the extent to which they endorsed each statement on a bipolar five-point rating scale (“1 = does not describe me well” to “5 = describes me well”), resulting in summed scores for each subscale as the dependent variables. For the IRI subscales, internal reliability ranged from 0.71 to 0.77, and test-retest reliability was indicated as ranging from 0.62 to 0.71 [48].

#### 2.3.2. Behavioral Empathy (Multifaceted Empathy Test)

Behavioral empathy was assessed with the Multifaceted Empathy Test (MET). The MET [49] involves the presentation of 40 positively and negatively valanced pictures of humans in emotionally charged situations. The participants had to choose out of four alternative adjectives, the one describing best the presented person’s emotional state (cognitive empathy). A library of emotional adjectives was presented throughout test administration to make sure that choosing the correct answer (scoring one point each) was not hampered by a lack of familiarity with any of the mental state descriptors. The number of correct responses served as the dependent variable, computed separately for positive and negative emotional states (minimum: 0, maximum: 20). Furthermore, in an implicit emotional empathy condition, participants were instructed to rate on a bipolar nine-point rating scale (“1 = not at all” to “9 = very strongly”) how strongly they personally responded to the presented pictures (personal affective involvement). In an explicit emotional empathy condition, participants indicated, using the same 9-point-rating scale, how strongly they were concerned for the presented person’s feelings (“empathic concern”). The summed rating scores, calculated separately for positive and negative valence, represented the dependent variables for explicit emotional empathy (empathic concern) and implicit emotional empathy (personal affective involvement) conditions (minimum: 20, maximum: 180).

Each of the three conditions involved the presentation of all 40 photographs, yielding 120 trials in total. Pictures were administered in blocks of subsequently presented 10 items belonging to the same condition, each being introduced by one of the following questions: “how does the person feel?” (cognitive empathy), “how strongly are you concerned for the person?” (empathic concern), “how emotionally aroused are you by the picture?” (personal affective involvement). The order of pictures was pseudorandomized within conditions. The presentation of pictures was controlled manually by the experimenter without any time constraints. Participants gave their responses verbally, with no time constraints, with the responses being recorded by the experimenter.

#### 2.3.3. Social Problem-Solving Fluency Task

To assess social problem-solving, a German abbreviated version [50] of the Social Problem-Solving Fluency Task [22] was used. The task assessed the ability to detect and interpret awkwardness in written hypothetical real-life problematic social situations, the discomfort experienced in those situations, the capacity to freely generate appropriate solutions as well as the ability to recognize the optimal solution when presented among a range of alternative less appropriate strategies. Two shortened parallel versions, A and B, containing five of the ten scenarios of the original English version each, were used in this study to limit the duration of testing. The shortened versions had previously been administered to patients with depression [50], alcohol use disorder [51] and attention deficit hyperactivity disorder [52]. Half of the PCNSL patients and controls, respectively, were either administered version A or B in a counterbalanced fashion. This procedure ensured that in both groups, equal numbers of participants were assessed with the two versions to limit the possibility that any group differences were due to potential differences in scenario difficulty of the two parallel versions. Participants had to respond verbally to a series of questions presented along with the scenarios. Answers were audio-recorded and subsequently transcribed and scored according to the rules specified in the original manual [22]. The first two questions were control questions assessing whether participants generally understood the storyline, scoring 1 for correct and 0 for incorrect answers, which were converted into percentages of maximum possible summed scores (10 maximum for all scenarios in total).

To detect the ability to recognize awkwardness in social situations, participants were asked to indicate why the situation described in the scenario might be awkward for the main character. Depending on whether the awkward element in the scenario was detected, the response was either scored with 1 or 0 points. Summed scores were converted to percentages of the maximum possible score (five for all scenarios in total) and used as a dependent variable. Afterward, participants had to rate the degree of awkwardness on a scale ranging from 0% (not at all awkward) to 100% (extremely awkward). The mean score of subjective awkwardness computed across all five scenarios was used as the dependent variable. Furthermore, within one minute, participants had to come up with as many good suggestions as they could think of for how the main character could address the difficult social situation described in the scenarios. Solutions were categorized according to their social sensitivity and practical effectiveness. Optimal solutions were considered to solve the problem both in a socially sensitive and practically effective (SP) manner. Accordingly, solutions could be judged as being only socially sensitive (S) or practically effective (P) or neither socially sensitive nor practically effective (N). Scoring of solutions was conducted by M.P. according to the original manual [22]. The manual provides detailed scoring guidelines with several examples illustrating different possible responses and their scoring for each social situation, thereby reducing the need for individual judgment. The agreement of two independent raters (i.e., inter-rater reliability) reportedly was found to be as high as 95%, and internal consistency amounted to 0.67 [22]. The total number of solutions falling into the four categories (SP, S, P, N) summed across all five scenarios served as the dependent variable. Following the end of each scenario, participants had to select the best (SP) solution out of four alternatives, with only one corresponding to the SP alternative. The dependent variable consisted of the percentage of the maximum possible number of times (in total five) the SP alternative was selected as the appropriate alternative across scenarios.

Example scenario:

“Mark is organizing a concert for charity. His friend loves singing but cannot sing in tune. His friend offers to perform a solo in the concert.”

1. Control question: Who is organizing the concert?
2. Social problem-solving questions:
  - Why may the situation be awkward for Mark?
  - How awkward a situation is it for Mark, out of 100%?
  - What could Mark do in this situation? Suggest as many good ideas as you can for dealing with the situation. You have one minute.
  - SP example: Explain tactfully that he needs professionals.
  - S example: Let his friend sing a solo in the concert.
  - P example: Tell his friend that he cannot sing in tune.
  - N example: Cancel the concert.

## 3. Procedure

Testing for this study took place in a particular, separate, quiet room used for neuropsychometric testing only. Thirty-one patients were recruited for study participation during their neurological routine follow-up, and 12 patients were additionally contacted and enrolled for the study. None suffered from a relapse until their next regular neurological routine follow-up. Both patients and healthy controls were tested in the same room, where only the investigator was present. Assessment procedures were identical for PCNSL patients and healthy controls. After providing written informed consent, the pre-assessment questionnaire was filled in by the participants. Following this, the MET, the Social Problem-Solving Fluency Task and the IRI questionnaire were administered. Afterward, depressive symptoms, verbal fluency and the estimated overall intelligence score were assessed. Participants had the possibility to take a break. The duration of testing was approximately two hours.

## 4. Data Analysis

Statistical analyses were carried out using SPSS statistics software (version 21). Group differences were analyzed using *t*-tests and repeated-measures ANOVAs where appropriate (Table 3). In the ANOVAs, the group was always considered as between-subject factor and, e.g., fluency condition (Regensburg verbal fluency test), subscale (IRI), valance (MET) or solution category (Social Problem-Solving Fluency Task) as the respective within-subject factor. The significance level was set to 0.05. Significant interactions were analyzed using post hoc Bonferroni-corrected *t*-tests. Differences in the gender ratio were analyzed with the *χ*^2^-test. Parametric tests were used as they have been found to be robust to violations of normality and equal variances in group comparisons involving equal sample sizes (*n* = 43) [53]. However, to prove whether the effects were robust additional nonparametric analyses were conducted. Greenhouse–Geisser adjustment was used when sphericity was violated. According to this correction, statistical parameters and degrees of freedom were adjusted. If patients and healthy controls significantly differed concerning neuropsychological background measures or estimated overall intelligence scores, these variables were selected post hoc as potential confounders of sociocognitive performance and additional analyses (i.e., correlations and analyses of covariance, ANCOVAs) were performed to control for these differences.

## 5. Results

### 5.1. Social Cognition Measures

#### 5.1.1. Dispositional Empathy

A repeated-measures ANOVA involving the four subscales (within-subject factor) of the self-report measure IRI and group (between-subject factor) revealed a significant interaction of subscale and group (*F*(2.7,230.7) = 3.869, *p* = 0.012, *η*^2^ = 0.044). Post hoc *t*-tests revealed that PCNSL patients scored higher on the personal distress subscale (*t*(84) = −2.302, *p* = 0.024, *d* = 0.496) as a component of emotional empathy relative to healthy controls. PCNSL patients presented marginally significantly (*t*(84) = 1.988, *p* = 0.050, *d* = 0.429) lower scores on the perspective-taking subscale (Table 4) as an aspect of cognitive empathy. Both group differences did not survive an applied Bonferroni-correction (*p* corrected < 0.0125). Group differences on empathic concern (emotional empathy) and fantasy (cognitive empathy) scores were not significant (both *p-*values ≥ 0.647).

Additionally, a significant main effect of IRI subscale was found (*F*(2.7,230.7) = 56.034, *p* < 0.001, *η*^2^ = 0.400). Post hoc Bonferroni-corrected *t*-tests computed across all participants revealed overall higher perspective-taking scores as compared to personal distress and fantasy scores (both *p-*values < 0.001), lower personal distress scores as compared to empathic concern and fantasy scores (both *p-*values ≤ 0.001), as well as higher empathic concern scores as compared to fantasy scores (*p* < 0.001). There was no main effect on the group (*p* = 0.796).

#### 5.1.2. Behavioral Empathy

**Figure 1:**
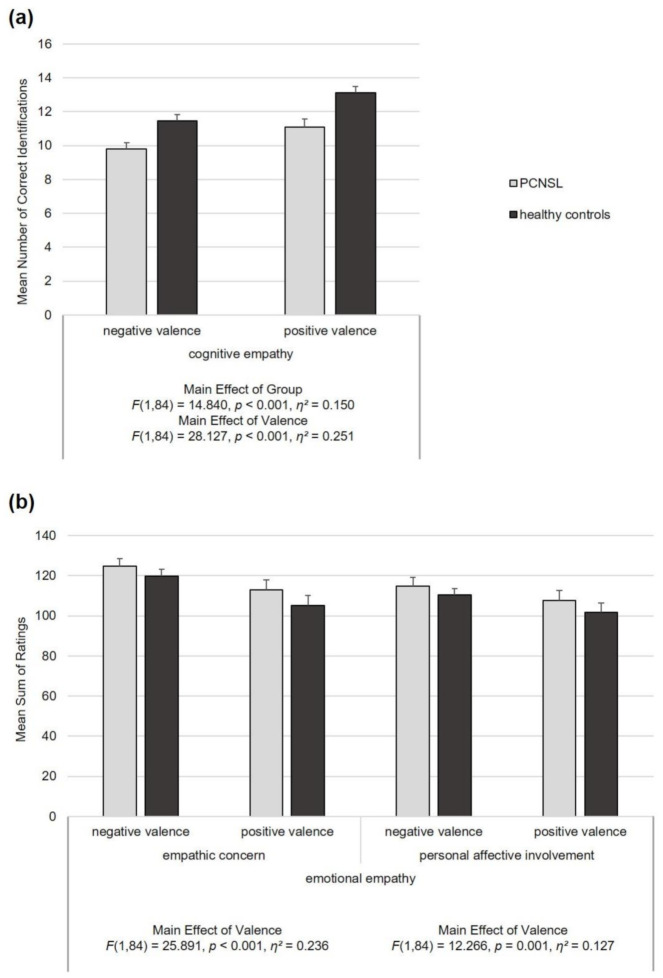
Behavioral cognitive and emotional empathy, as assessed with the Multifaceted Empathy Test, presented for patients with primary central nervous system lymphoma (PCNSL) and healthy controls. The figure displays group mean scores and standard errors of the mean (error bars). (**a**) Cognitive empathy, i.e., the ability to cognitively understand another person’s feelings. (**b**) Emotional empathy, i.e., the ability to vicariously experience and respond to another person’s emotional state (feeling concerned and feeling emotionally affected in response to another person’s emotional state).

Repeated-measures ANOVAs were also computed to analyze performance on the MET separately for cognitive empathy, empathic concern and personal affective involvement, with the latter two representing aspects of emotional empathy (Figure 1). In each analysis, group (between-subject) and valence (within-subject) were considered as factors. The analysis of cognitive empathy revealed an overall significant main effect of group (*F*(1,84) = 14.840, *p* < 0.001, *η*^2^ = 0.150) with a lower number of correct emotion identifications in the patients (*p* < 0.001) as well as an overall significant effect of valence (*F*(1,84) = 28.127, *p* < 0.001, *η*^2^ = 0.251) reflecting overall better recognition of positive relative to negative emotional states (*p* < 0.001). The interaction was not significant (*p* = 0.480). Concerning empathic concern (*F*(1,84) = 25.891, *p* < 0.001, *η*^2^ = 0.236) and personal affective involvement (*F*(1,84) = 12.266, *p* = 0.001, *η*^2^ = 0.127) an overall significant effect of valence occurred with overall higher scores for negative emotional states for both dimensions of emotional empathy (both *p-*values ≤ 0.001). Regarding empathic concern and personal affective involvement no significant effects of group and no significant interactions of group and valence emerged (all *p-*values ≥ 0.236).

#### 5.1.3. Social Problem-Solving Fluency Task

Group comparison revealed that PCNSL patients and healthy controls did not differ regarding their performance on the control questions (*p* = 0.118) assessing general understanding of the storyline. Patients rated the degree of awkwardness experienced in the social situation depicted in the story on the same level as healthy controls did (*p* = 0.964) but identified the awkward elements in the depicted scenarios significantly less often accurately than healthy controls (*t*(68.2) = 3.764, *p* < 0.001, *d* = 0.812), Table 4.

**Figure 2:**
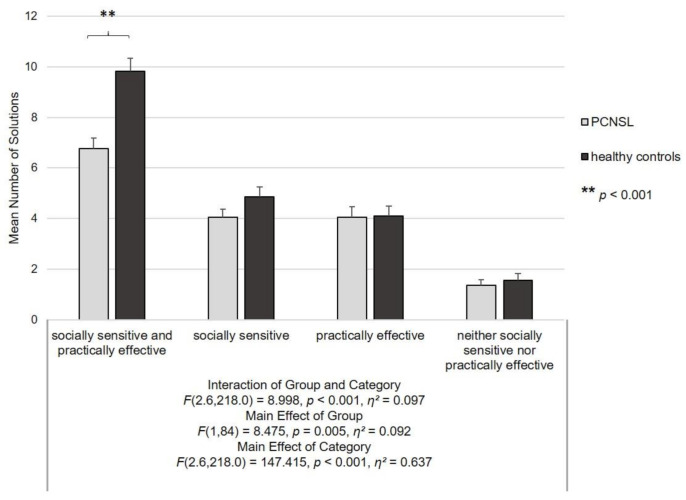
Social problem-solving fluency as assessed with the Social Problem-Solving Fluency Task. Differences between primary central nervous system lymphoma (PCNSL) patients and healthy controls concerning the number of problem solutions given within one minute for each solution category (socially sensitive and practically effective, merely socially sensitive, merely practically effective and neither socially sensitive nor practically effective). The significant difference in the number of socially sensitive and practically effective solutions as revealed by post hoc Bonferroni-corrected *t*-tests is marked by **. The figure displays the group mean scores and standard errors of the mean (error bars).

A repeated-measures ANOVA was computed to analyze performance on solution fluency (freely generated problem solutions) (Figure 2) with group (between-subject) and category (within-subject) considered as factors. The analysis revealed a significant interaction of group and category (*F*(2.6,218.0) = 8.998, *p* < 0.001, *η*^2^ = 0.097). In post hoc Bonferroni-corrected *t*-tests patients and healthy controls did not differ regarding the number of S, P and N solutions (all *p*-values ≥ 0.125). By contrast, PCNSL patients generated significantly fewer optimal SP solutions (*t*(84) = 4.577, *p* < 0.001, *d* = 0.987) as compared to healthy controls (*p* corrected < 0.0125 after application of Bonferroni-correction), Figure 2.

Additionally, significant main effects of both group (*F*(1,84) = 8.475, *p* = 0.005, *η*^2^ = 0.092) and category (*F*(2.6,218.0) = 147.415, *p* < 0.001, *η*^2^ = 0.637) occurred. Post hoc Bonferroni-corrected *t*-tests computed across all participants revealed an overall higher number of solutions for the healthy control group (*p* = 0.005) and overall lower number of N as compared to SP, S and P solutions (all *p-*values < 0.001) as well as overall higher number of SP as compared to S and P solutions computed across all participants (both *p*-values < 0.001).

PCNSL patients recognized the SP solution significantly less often correctly (*t*(84) = 2.672, *p* = 0.009, *d* = 0.576) when it was presented amidst less optimal strategies as compared to healthy controls (Table 4).

### 5.2. Additional Analyses to Control for Neuropsychological Background Measures and Estimated Overall Intelligence Scores

Since patients and healthy controls differed significantly on BDI scores and verbal fluency, correlations according to Pearson were computed between indicators of sociocognitive performance and these variables in the patient group only. Due to the number of correlations involved, the significance level for these analyses was set to a stricter value of 0.01. If significant correlations were detected, exploratory ANCOVAs were performed. BDI scores (*p* = 0.002) and overall verbal fluency scores (all *p-*values ≤ 0.009) were significantly correlated with indicators of sociocognitive performance. The group differences on cognitive empathy and relevant measures of the Social Problem-Solving Fluency Task (i.e., the interaction of group and category) remained significant when statistically controlling (ANCOVAs) separately for confounding effects of BDI and verbal fluency scores (*n* = 43, Results S1). PCNSL patients and healthy controls differed significantly on estimated overall intelligence scores, which are sometimes referred to as an assessment of premorbid cognitive abilities. Therefore, all analyses were repeated with the estimated overall intelligence scores as a covariate. The interaction of subscale and group in the analysis of the IRI was abolished (*p* = 0.203), and the group difference on recognizing the optimal SP solution amidst less optimal strategies failed to reach significance (*p* = 0.051) when statistically controlling for estimated overall intelligence scores (ANCOVAs). By contrast, including estimated overall intelligence scores as a covariate did not change the group differences for the remaining measures of the Social Problem-Solving Fluency Task and concerning behavioral cognitive empathy (Results S1).

### 5.3. Additional Analyses Regarding Different Subgroups of Patients

Focal neurological or neuropsychological symptoms were present in 10 patients (Table S1). When repeating the analyses after excluding these patients and their respective paired matched healthy controls, the result pattern for cognitive empathy and social problem-solving remained stable (Results S2, Table S2). Furthermore, excluding patients who suffered from a cerebral tumor relapse did not change the result pattern concerning sociocognitive performance (Results S3, Table S3). When comparing patients with tumor resection or open biopsy (*n* = 15) and those without (*n* = 28), no significant differences in sociocognitive performance emerged (Results S4, Table S4). When comparing patients having received high-dose chemotherapy followed by autologous stem cell transplantation (HDASCT) for consolidation and those, who did not, only for the detection of awkwardness and the subjective degree of awkwardness, significant group differences occurred with patients having received HDASCT performing better (Results S5, Table S5). Furthermore, for empathy and for the ability to freely produce and merely recognize appropriate solutions for interpersonal situations, no gender differences were found (Results S6, Tables S6 and S7).

### 5.4. Additional Non-Parametric Statistical Analyses

To prove whether the effects were robust non-parametric analyses for all sociocognitive measures were computed additionally. Only for the IRI subscale “perspective-taking”, the previously marginally significant group difference (*p* = 0.050) was abolished when using non-parametric tests (*p* = 0.126). However, it must be kept in mind that this group difference also did not withstand Bonferroni-correction when using parametric tests. The result pattern for all other sociocognitive measures was the same when using non-parametric or parametric statistical methods (Results S7, Table S8).

## 6. Discussion

While some previous studies have addressed social cognition in brain tumor patients in general, to the best of our knowledge, the present study is the first to provide a systematic and fine-grained analysis of empathy and social problem-solving in a group of PCNSL patients. Although a substantial fraction of these patients may experience long-term survival due to chemotherapy alone with preservation of neurological and neurocognitive function, as reported previously [8], cognitive empathy and social problem-solving abilities were impaired in the present study.

When discussing the results, some methodological aspects must be considered. First, hypothetical social situations were used, although it is remarkable that a selective solution generation deficit in PCNSL patients was observed even under these conditions. Second, it cannot be ruled out that impairments were already present before treatment. Third, the patients were not subjected to a comprehensive formal neuropsychological assessment to spare them additional testing after having undergone a two-hour assessment of sociocognitive function. Nevertheless, none of the patients suffered from cognitive impairment preventing independent living as reported to the treating physician. Fourth, increased depression scores and impaired overall verbal fluency in the patients represent potential confounders. However, when statistically controlling for these confounders, relevant group differences regarding sociocognitive performance remained significant (Results S1). The estimated overall intelligence score of 80 was meant to represent an inclusion criterion. Nevertheless, group differences on estimated overall intelligence scores, which are sometimes referred to as an assessment of premorbid cognitive abilities, might have influenced sociocognitive performance. When statistically controlling for estimated overall intelligence scores, the group differences in cognitive empathy and social problem-solving fluency remained significant (Results S1). This rather suggests that group differences on these sociocognitive measures were not (solely) driven by differences in premorbid estimated overall intelligence scores, although an influence cannot be excluded completely. One-third of our patients had undergone resection, which is possibly associated with postoperative neurological morbidity [3]. In addition, seven patients had received HDASCT for consolidation, which recently was discussed to be associated with delayed neurotoxicity [54]. To take the heterogeneity of our patient sample into account, we addressed this point (Results S4 and S5) and did not find relevant differences between these subgroups (patients who had undergone resection versus those who did not, and patients having received HDASCT for consolidation versus those, who did not). However, these analyses were of exploratory character only. In our opinion, everyday clinical practice is presumably mirrored best by including patients with heterogeneous treatment courses.

A series of clinically relevant results emerged from the present study: Significantly decreased behavioral cognitive empathy was observed in PCNSL patients as compared to healthy controls. Behavioral emotional empathy was descriptively but not statistically significantly enhanced in the patients. Regarding the Social Problem Solving Task, PCNSL patients and healthy controls did not differ on control questions indicating that the general understanding of the storyline was not compromised. Since both PCNSL patients and healthy controls scored at > 93% correct, we cannot completely exclude the possibility that statistical analyses of control questions might be restrained by ceiling effects. Nevertheless, overall understanding of the storyline was ensured according to the original manual [22]. Therefore, it is presumed that potential ceiling effects within the control questions did not confound the interpretation of the relevant indices of social problem-solving in the same task, particularly given that no ceiling effects were observed for these social problem-solving measures that would invalidate their results. PCNSL patients rated the degree of discomfort of an awkward situation on a comparable level as healthy controls did but were less able to accurately identify the element that evokes awkwardness. Since those differences remained after correction for estimated overall intelligence was applied, they presumably represent not (solely) a result of different levels of comprehension. Concerning the detection of awkwardness and the subjective degree of awkwardness, a gender difference was found for PCNSL patients only, such that male patients performed more poorly. This is in accordance with findings of a generally decreased emotional responsiveness in males [55]. However, regarding all other sociocognitive measures, no gender differences occurred. Both the production of optimal solutions for interpersonal conflicts and the mere recognition of these among less optimal strategies were impaired in PCNSL patients. These sociocognitive impairments were present both in the whole patient group (*n* = 43) as well as in subgroups of patients (i.e., when excluding patients with neurological symptoms or patients having suffered from a cerebral relapse, who underwent two lines of treatment, Results S2 and S3). This strengthened our notion of a specific sociocognitive deficit that may develop in PCNSL patients independently of clinical characteristics and neurological impairment. Since these results were confirmed with non-parametric analyses (Results S7), we assume that our effects are robust.

In preceding studies, behavioral cognitive empathy, assessed by the “Reading the Mind in the Eyes Test” (RMET), was impaired in a heterogeneous group of preoperative brain tumor patients [39]. While in the RMET, only the eye region is presented, the MET allowed to determine whether social contextual information may improve performance and to assess behavioral emotional empathy. However, impaired cognitive empathy for both positive and negative mental states was evident in PCNSL patients. When excluding PCNSL patients with focal neurological or neuropsychological deficits from the analyses, PCNSL patients additionally showed significantly increased behavioral emotional empathy compared to healthy controls, which was only a descriptive trend in the whole patient group. Increased emotional empathy may impair accurate information processing necessary for an accurate understanding of the interaction partner’s mental states (i.e., cognitive empathy) in an emotionally charged situation.

Concerning self-reported empathy, personal distress as an emotional empathy component was increased, and perspective-taking as a cognitive component was decreased in PCNSL patients relative to healthy controls. Hence, self-reported empathy components mirrored the results for the behavioral empathy measures. However, it must be kept in mind that these self-reported differences did not withstand corrections for multiple comparisons.

Previous research on social problem-solving is scarce. An early study reported comparable ratings of PCNSL patients on the Problem-Solving Inventory as compared to normative data [9]. However, this study is limited in its generalizability due to the small number of patients who were eligible for assessment, use of self-report measures only and lack of a healthy control group. By contrast, employing a scenario-based task involving real-life social situations, we did observe specifically impaired performance of PCNSL patients relative to healthy controls. Difficulties in the understanding of social signals (cognitive empathy) may prevent patients from appropriately responding to issues with interaction partners (social problem-solving). This may contribute to the discomfort experienced in social situations and consequently to avoidance of those situations. Evading social interactions reduces a patient’s engagement in social groups and leads to social isolation [56], long recognized to negatively influence QoL [57].

In the present study, depression scores were significantly negatively associated with social problem-solving fluency. Controlling for depression, however, did not change group differences on sociocognitive performance, i.e., PCNSL patients were still impaired in providing optimal solutions for social problems. While some studies on other neurological diseases documented a negative association between depression and empathy [58], others did not observe such correlations [59]. In studies involving patients with major depression, a reduced ability to reason about other people’s mental states [60] was reported. Furthermore, in a study using the same Social Problem-Solving Fluency Task, depressed patients generated fewer solutions that were socially sensitive and practically effective or merely socially sensitive. In contrast to the present findings in PCNSL patients, the mere recognition of optimal solutions among alternatives was intact in patients with major depressive disorder [50].

Concerning the mechanisms leading to sociocognitive impairments, one can assume that motivational processes may potentially influence patients’ performance rather than patients’ abilities per se. PCNSL patients may have less energy and inclination to care about other peoples’ thoughts and feelings after having gone through a potentially life-threatening disease and intensive treatment. On the other hand, being diagnosed with and treated for a brain tumor, however, is a potentially traumatic experience accompanied by fundamental psychological changes [61]. Since sociocognitive impairments were found in post-traumatic stress disorder [25], one can speculate that sociocognitive impairments in PCNSL patients having been treated with intensive chemotherapy for many weeks may be due to a long-lasting confrontation with a potentially lethal disease and not due to clinical characteristics of the disease or treatment themselves.

Further research should include ecologically valid measures of patients’ social well-being or role functioning. Among other aspects, patients’ subjective awareness of their level of social functioning and whether other people noticed social deficits should be assessed. Moreover, standardized measures of QoL and resilience should be included in further studies to support the understanding of associations between social cognition and everyday functioning in brain tumor patients. Furthermore, further research on sociocognitive abilities should additionally address patients’ level of functioning in society (e.g., return to work [42]) and should specifically capture other relevant aspects of patients’ social functioning (e.g., hobbies, sporting activities, gathering with friends and family) in a prospective manner.

It is important to increase the awareness of treating physicians to “real-life” social problems in cured PCNSL patients that may be overlooked in clinical practice. Our results highlight that PCNSL patients differ from healthy controls in their social abilities even in the absence of (residual) disease itself. These problems may potentially account for difficulties in reintegration as observed in PCNSL survivors [42] and should be considered therapeutically. Successful interventions, which help patients to develop adequate strategies for the solution of social problems, have already been positively evaluated for patients with acquired brain injuries or psychiatric symptoms [62] as well as for pediatric brain tumor survivors [63]. With advances in treatment and increased numbers of cancer survivors, specific significance must be paid to the psychological and social impact of cancer as a “chronic” disease as it affects not only the patients’ but also the caregivers’ QoL.

## 7. Conclusions

This study demonstrates that PCNSL patients differ from healthy controls in their sociocognitive abilities even in the absence of (residual) disease itself. Since sociocognitive impairments may evoke difficult interpersonal situations, they may represent an additional burden affecting patients’ and caregivers’ QoL. Additional attention should be paid to those potential impairments.

## Figures and Tables

**Table 1:** Clinical characteristics.

PCNSL Patients					
					Total
Neuropathology	Diffuse large B-cell lymphoma	Other			
	43	0			43
Tissue diagnosis by	Tumor resection	Open biopsy	Stereotactic biopsy	CSF ^1^ cytology	
	14	1	26	2	43
First-line treatment	HDMTX ^2^-based polychemoimmunotherapy, followed by intensified conventional chemotherapy plus intraventricular treatment for consolidation	HDMTX-based polychemoimmunotherapy, followed by intensified conventional chemotherapy without intraventricular treatment for consolidation	HDMTX-based polychemoimmunotherapy, followed by HDASCT ^3^ for consolidation	HDMTX-based chemotherapy with temozolomide alone for consolidation	
	32	3	7	1 ^4^	43
Tumor relapse	No		Yes		
	37		6 (5 cerebral, 1 ocular only, no systemic relapse)		43
Treatment at relapse	Two patients were treated with HDASCT, and three patients were treated with intensified conventional chemotherapy. All five patients received complete remission after salvage treatment. The patient, who suffered from an ocular relapse, received radiation with 36 + 14 Gray fractionized to 5 × 2 Gray to two-thirds of the posterior eye bulb.				

^1^ cerebrospinal fluid; ^2^ high-dose methotrexate; ^3^ high-dose chemotherapy followed by autologous stem cell transplantation; ^4^ according to the patient’s wish.

**Table 2:** Demographic data, estimated overall intelligence, verbal fluency and severity of depressive symptoms of primary central nervous system lymphoma (PCNSL) patients and healthy controls. The table presents absolute values or mean scores with standard deviations in brackets as well as test statistics.

Data	PCNSL Patients	Healthy Controls	Test Statistics
*N*	43	43	
Median age at testing (years)	65 (range 37–83)	66 (range 35–80)	n.s.
Gender (female:male)	21:22	21:22	n.s.
Years of school	10.56 (2.21)	11.38 (1.85)	n.s.
Years of education	14.92 (3.70)	15.63 (3.30)	n.s.
Estimated overall intelligence	113.63 (13.82)	121.67 (12.68)	*t*(82) = 2.780, *p* = 0.007, *d* = 0.607
Regensburg verbal fluency test (number of words within one minute)			
Phonematic verbal fluency	10.86 (4.05)	11.70 (4.21)	Main effect of group *F*(1,84) = 6.077, *p* = 0.016, *η*^2^ = 0.067 main effect of condition *F*(2,168) = 229.433, *p* < 0.001, *η*^2^ = 0.732
Semantic verbal fluency one category	20.86 (5.79)	23.56 (5.16)	
Semantic verbal fluency category switch	13.37 (3.28)	15.07 (2.73)	
Beck Depression Inventory score	10.30 (9.13)	5.16 (3.71)	*t*(55.5) = −3.420, *p* = 0.001, *d* = 0.738

**Table 3:** Measures and statistical methods used.

Data and Measures	Statistical Method
Age	*t*-test
Gender	*χ*^2^-test
Years of school	*t*-test
Years of education	*t*-test
German multiple-choice vocabulary intelligence test	*t*-test
Regensburg verbal fluency test	Repeated-measures analysis of variances
Beck Depression Inventory score	*t*-test
Interpersonal Reactivity Index	Repeated-measures analysis of variances
Multifaceted Empathy Test	Repeated-measures analysis of variances
Social Problem-Solving Fluency Task, control questions	*t*-test
Social Problem-Solving Fluency Task, detection of awkwardness	*t*-test
Social Problem-Solving Fluency Task, subjective degree of awkwardness	*t*-test
Social Problem-Solving Fluency Task, solution fluency	Repeated-measures analysis of variances
Social Problem-Solving Fluency Task, selection of optimal alternatives	*t*-test

**Table 4:** Performance of primary central nervous system lymphoma (PCNSL) patients and healthy controls concerning self-reported empathy and social problem-solving. The table presents absolute values or mean scores with standard deviations in brackets as well as test statistics.

Performance Measure	PCNSL Patients	Healthy Controls	Test Statistics
*N*	43	43	
Interpersonal Reactivity Index			
Empathic concern	14.51 (2.75)	14.30 (2.48)	Interaction of subscale and group *F*(2.7,230.7) = 3.869, *p* = 0.012, *η*^2^ = 0.044 Main effect of subscale *F*(2.7,230.7) = 56.034, *p* < 0.001, *η*^2^ = 0.400
Personal distress	11.23 (3.34)	9.79 (2.39)	
Fantasy	12.02 (2.88)	12.30 (2.75)	
Perspective-taking	14.12 (2.64)	15.12 (1.98)	
Social Problem-Solving Task			
Control questions (mean percent correct)	93.95 (11.78)	97.09 (5.48)	n.s.
Detection of awkwardness (mean percent correct)	64.88 (27.55)	83.26 (16.29)	*t*(68.2) = 3.764, *p* < 0.001, *d* = 0.812
Subjective degree of awkwardness (mean rating percent)	72.78 (18.49)	72.63 (11.81)	n.s.
Selection of optimal (SP ^1^) alternatives (mean percent correct)	49.77 (20.64)	61.40 (19.71)	*t*(84) = 2.672, *p* = 0.009, *d* = 0.576

^1^ socially sensitive and practically effective.

## Data Availability

The data presented in this study are available on request from the corresponding author. The data are not publicly available to avoid the risk of patient identification. As primary central nervous system lymphomas represent a rare brain tumor entity the identity of patients may be inferred from personal demographic data.

## Supplementary Materials

The following are available online at https://www.mdpi.com/2072-6694/13/5/943/s1, Results S1: Additional Analyses to Control for Neuropsychological Background Measures and Estimated Overall Intelligence Scores in the Whole Patient Group (*n* = 43), Results S2: Changes of the Result Pattern when Excluding PCNSL Patients with Focal Neurological or Neuropsychological Symptoms from the Analyses, Results S3: Changes of the Result Pattern when Excluding PCNSL Patients Who Suffered from a Cerebral Tumor Relapse from the Analyses, Results S4: Differences of Sociocognitive Functions between Patients Who Had Undergone Resection and Those Who Did Not, Results S5: Differences of Sociocognitive Functions between Patients Having Received High-Dose Chemotherapy Followed by Autologous Stem Cell Transplantation for Consolidation and Those Who Did Not, Results S6: Gender Differences in Sociocognitive Performance, Results S7: Additional Non-Parametric Analyses, Text S1: Additional Patients’ Characteristics, Table S1: Patients with focal neurological or neuropsychological symptoms, Table S2: Demographic data, severity of depressive symptoms and performance concerning verbal fluency, self-reported and behavioral empathy and social problem-solving of a reduced subgroup of patients with primary central nervous system lymphoma (PCNSL) and healthy controls, Table S3: Demographic data, severity of depressive symptoms and performance concerning verbal fluency, self-reported and behavioral empathy and social problem-solving of a reduced subgroup of patients with primary central nervous system lymphoma (PCNSL) and healthy controls, Table S4: Severity of depressive symptoms and performance concerning self-reported and behavioral empathy and social problem-solving of patients with primary central nervous system lymphoma (PCNSL) having undergone resection or open biopsy and patients, who did not, Table S5: Severity of depressive symptoms and performance concerning self-reported and behavioral empathy and social problem-solving of patients with primary central nervous system lymphoma (PCNSL) having received high-dose chemotherapy followed by autologous stem cell transplantation for consolidation and those, who did not, Table S6: Demographic data, severity of depressive symptoms and performance concerning self-reported and behavioral empathy and social problem-solving of female and male PCNSL patients, Table S7: Demographic data, severity of depressive symptoms and performance concerning self-reported and behavioral empathy and social problem-solving of female and male healthy participants, Table S8: Non-parametric statistics concerning self-reported and behavioral empathy and social problem-solving of patients with primary central nervous system lymphoma (PCNSL) and healthy controls.
