# Supplementary material for: What Is on Your Mind? Impaired Social Cognition in Primary Central Nervous System Lymphoma Patients Despite Ongoing Complete Remission
Source: Cancers (Basel). 2021 Feb 24;13(5):943. doi: 10.3390/cancers13050943 (PMC7956780; doi:10.3390/cancers13050943)
Supplement: Supplementary file 1 [file cancers-13-00943-s001.pdf]

cancers

MDPI

Supplementary Material: What Is on Your Mind? Impaired Social Cognition in Primary Central Nervous System Lymphoma Patients Despite Ongoing Complete Remission

Milena Pertz, Thomas Kowalski, Patrizia Thoma and Uwe Schlegel

Text S1: Additional Patient's Characteristics

Six out of 43 primary central nervous system lymphoma (PCNSL) patients suffered from a tumor relapse that was only ocular in one patient. Two patients were treated at relapse with high-dose chemotherapy followed by autologous stem cell transplantation (HDASCT). In both patients time between completion of first-line treatment and tumor relapse was 27 months. Three patients, who suffered from a relapse 22, 120 and 137 months after first-line treatment, were treated with intensified conventional chemotherapy. Salvage treatment led to complete remission in all five patients. In one patient, an ocular relapse occurred seven months after first-line treatment. The patient received radiation with 36 + 14 Gray fractionized to 5 × 2 Gray to two-thirds of the posterior eye bulb.

Thirty-one patients were recruited for study participation during their neurological routine follow-up and 12 patients were additionally contacted and enrolled for the study. None suffered from a relapse until their next regular neurological routine follow-up. Healthy controls participated between October 2018 and March 2020 and were recruited per advertisements placed in a regional newspaper and via addressing acquaintances.

Of 43 patients, 10 presented with focal neurological or neuropsychological symptoms, as listed in their medical records (Table S1).

Table S1. Patients with focal neurological or neuropsychological symptoms.

Patient	Age at Participation	Gender	KPS	Symptom	Cause
Patient 1	60	female	60	cerebellar syndrome	resection of right frontal PCNSL with external ventricular drainage because of fourth ventricle compression and consecutive occlusive hydrocephalus
Patient 2	80	female	80	hemianopia to the left side	resection of right occipital PCNSL
Patient 3	83	male	80	hemianopia to the left side	resection of right occipital PCNSL
Patient 4	76	male	90	minor oculomotor disturbance	resection of PCNSL localized in the left cerebellar peduncle
Patient 5	74	female	90	minor oculomotor disturbance	resection of left cerebellar PCNSL
Patient 6	56	female	80	tetraparesis with preserved ability to walk	motor neuropathy, unrelated to PCNSL, i.e. no symptom caused by PCNSL treatment
Patient 7	65	male	70	cognitive impairment with psychomotor slowing	PCNSL localized left temporal, HDMTX-based polychemoimmunotherapy, followed by intensified conventional chemotherapy plus intraventricular treatment for consolidation, tumor relapse (corpus callosum and left putamen) treated with HDASCT
Patient 8	49	male	90	mild hemiparesis to the left side	PCNSL localized in right brainstem and right basal ganglia, HDMTX-based polychemoimmunotherapy, followed by HDASCT for consolidation
Patient 9	44	male	80	psychomotor slowing, impairment in	PCNSL localized right temporobasal and right occipital, HDMTX-based

				attention and verbal memory	polychemoimmunotherapy, followed by intensified conventional chemotherapy plus intraventricular treatment for consolidation
Patient 10	56	male	80	hemianopia to the right side	left parietal cerebral abscess following stereotactic biopsy with operative revision

KPS: Karnofsky Performance Scale score, PCNSL: primary central nervous system lymphoma, HDMTX: high-dose methotrexate, HDASCT: high-dose chemotherapy followed by autologous stem cell transplantation.

Results S1: Additional Analyses to Control for Neuropsychological Background Measures and Estimated Overall Intelligence Scores in the Whole Patient Group (n = 43)

Since patients and healthy controls differed significantly on Beck Depression Inventory (BDI) scores and verbal fluency correlations according to Pearson were computed between indicators of sociocognitive performance (Interpersonal Reactivity Index (IRI) subscales, Multifaceted Empathy Test (MET) cognitive empathy scores, MET empathic concern scores, MET personal affective involvement scores (all MET scores aggregated across both valences), detection of awkwardness, subjective degree of awkwardness, number of socially sensitive and practically effective (SP), socially sensitive (S), practically effective (P), and neither socially sensitive nor practically effective (N) solutions, selection of optimal solutions in the Social Problem Solving Fluency Task) and those variables in the patient group only. Since primary central nervous system lymphoma (PCNSL) patients and healthy controls differed in verbal fluency, irrespective of category (main effect of group), a mean score was calculated for all phonematic and semantic verbal fluency conditions and entered into the correlational analyses. Concerning verbal fluency, correlations were computed with the number of SP, S, P and N solutions in the Social Problem Solving Fluency Task to specifically control for overall differences in verbal fluency which is likely to play a role for performance on this task. Due to the number of correlations involved, the significance level for these analyses was set to a stricter value of 0.01. If significant correlations were detected exploratory analyses of covariance (ANCOVAs) were performed.

BDI scores were significantly correlated with the number of SP solutions in the Social Problem Solving Fluency Task (r = −0.449, p = 0.002). The verbal fluency mean score was significantly associated with the number of SP (r = 0.505, p = 0.001), S (r = 0.395, p = 0.009) as well as P solutions (r = 0.420, p = 0.005) in the Social Problem Solving Fluency Task. There were no other significant correlations (in terms of the stricter p-value of 0.01) between indicators of sociocognitive performance and BDI scores or verbal fluency mean scores in PCNSL patients (all p-values ≥ 0.044).

To additionally rule out that group differences in social cognition were fully explained by differences in BDI scores or verbal fluency, exploratory ANCOVAs were performed with the respective variable as a covariate. This changed the result pattern as follows: When including the verbal fluency mean score in the analysis of solution fluency in the Social Problem Solving Task the significant main effect of category was abolished (p = 0.522) and the main effect of group remained only marginally significant (F(1,83) = 3.839, p = 0.053, η^2 = 0.044). By contrast the significant interaction of category and group remained significant (F(2.6,213.2) = 6.656, p = 0.001, η^2 = 0.074) when including the verbal fluency mean score as a covariate. Furthermore, when including BDI scores as a covariate in the analysis of solution fluency in the Social Problem Solving Task, the result pattern of the main analysis did not change, i.e. the main effect of both group (F(1,83) = 4.711, p = 0.033, η^2 = 0.054) and category (F(2.6,213.4) = 103.577, p < 0.001, η^2 = 0.555) as well as the interaction of group and category (F(2.6,213.4) = 4.737, p = 0.005, η^2 = 0.054) remained significant. Consequently, it was concluded that group differences concerning the number of optimal

SP solutions in the Social Problem Solving Task were not (solely) driven by group differences on BDI scores or verbal fluency alone.

Patients and healthy controls differed significantly on estimated overall intelligence scores which are sometimes referred to as an assessment of premorbid cognitive abilities. To exclude the possibility that between-group premorbid intelligence differences were driving the differences in sociocognitive performance all analyses were repeated including the estimated overall intelligence scores as covariate. This changed the result pattern as follows: When including the estimated overall intelligences scores in the analysis of dispositional empathy (IRI) the main effect of subscale (p = 0.248) and the interaction of subscale and group (p = 0.203) were abolished. In this vein, it has to be kept in mind, that group differences represented within this interaction did not withstand an applied Bonferroni-correction in the main analyses either. When including the estimated overall intelligences scores in the analysis of behavioral cognitive empathy (MET) the main effect of valence was abolished (p = 0.371). By contrast, the significant main effect of group remained significant ($F(1,81)$ = 7.510, p = 0.008, η^2 = 0.085). Regarding empathic concern (MET), the significant main effect of valence was abolished (p = 0.192). Likewise, in the analysis of solution fluency in the Social Problem Solving Task the main effect of category was abolished (p = 0.530). By contrast, the interaction of group and category in the Social Problem Solving Task ($F(2.6,213.7)$ = 5.383, p = 0.002, η^2 = 0.062) and the main effect of group ($F(1,81)$ = 5.528, p = 0.021, η^2 = 0.064) remained significant when including the estimated overall intelligence scores as a covariate. In the analysis of recognition of the SP alternative amidst less optimal strategies the group difference failed to reach significance narrowly ($F(1,81)$ = 3.926, p = 0.051, η^2 = 0.046). The result pattern of all other assessed measures (i.e. MET personal affective involvement, Social Problem Solving Task control questions, detection of awkwardness, subjective degree of awkwardness) did not change when including estimated overall intelligence scores as a covariate. This suggests that group differences on sociocognitive performance (i.e. cognitive empathy and relevant measures of the Social Problem Solving Fluency Task) were not (solely) driven by between-group premorbid intelligence differences.

Results S2: Changes of the Result Pattern when Excluding PCNSL Patients with Focal Neurological or Neuropsychological Symptoms from the Analyses

Ten out of 43 patients had focal neurological or neuropsychological symptoms (e.g. psychomotor slowing), as listed in Table S1. To rule out that sociocognitive impairment in the whole PCNSL group (n = 43) was caused by focal neurological or neuropsychological impairment, we repeated our analyses after excluding the 10 patients and their respective 10 matched healthy controls (n = 33) since we used a pair-matching procedure.

This changed the result pattern as follows: In the repeated-measures analysis of variances (ANOVA) to analyze number of words in the Regensburg verbal fluency test, with group as between-subject and fluency condition as within-subject factor, the significant main effect of group was abolished (p = 0.098). PCNSL patients and healthy controls (each n = 33) were not significantly different on their overall verbal fluency. In the repeated-measures ANOVA involving the four IRI subscales (within-subject factor) and group (between-subject factor) the interaction of subscale and group was only marginally significant in the subgroups of 33 participants (p = 0.060). When considering empathic concern and personal affective involvement, as assessed with the MET, for both dimensions of emotional empathy, a significant main effect of group was found that was not present, when considering 43 PCNSL patients and healthy controls. Concerning empathic concern ($F(1,64)$ = 5.043, p = 0.028, η^2 = 0.073) and personal affective involvement ($F(1,64)$ = 4.183, p = 0.045, η^2 = 0.061) PCNSL patients scored significantly higher as compared to healthy controls irrespective of emotional valence. In the repeated-measures ANOVA to analyze performance on solution fluency in the Social Problem Solving Fluency Task with group (between-subject) and category (within-subject) as factors the main effect of group was only marginally significant (p = 0.070) when considering 33 PCNSL patients and their

respective controls. By contrast, the main effect of category ($F(2.6;164.3) = 116.814$, $p < 0.001$, $\eta^2 = 0.646$) as well as the interaction of category and group ($F(2.6;164.3) = 6.668$, $p = 0.001$, $\eta^2 = 0.094$) were still present in the smaller subgroups ($n = 33$) reflecting significantly decreased performance of patients when producing SP solutions. See Table S2 for the full report of the statistical analysis. The result pattern of all other assessed measures did not change when excluding 10 patients and their respective matched healthy controls from the analyses.

Table S2. Demographic data, severity of depressive symptoms and performance concerning verbal fluency, self-reported and behavioral empathy and social problem solving of a reduced subgroup of patients with primary central nervous system lymphoma (PCNSL) and healthy controls. The table presents absolute values or mean scores with standard deviations presented in brackets as well as the test statistics.

			**PCNSL Patients**	**Healthy Controls**	**Test Statistics**
N			33	33	
Median age at testing (years)			66 (range 37-82)	67 (range 35-80)	$t(64) = -0.022$, $p = 0.982$
Gender (female:male)			17:16	17:16	$\chi^2(1) = 0.0$, $p = 1.0$
Years of school			10.64 (2.33)	11.47 (1.94)	$t(61.9) = 1.580$, $p = 0.119$
Years of education			14.85 (3.74)	15.62 (3.26)	$t(64) = 0.895$, $p = 0.374$
Estimated overall intelligence			114.79 (14.02)	121.70 (12.45)	$t(64) = 2.117$, $p = 0.038$, $d = 0.521$
German Regensburg verbal fluency test (number of words within one minute)		Phonematic verbal fluency	11.24 (3.90)	12.27 (3.69)	1) main effect of condition $F(2,128) = 202.853$, $p < 0.001$, $\eta^2 = 0.760$
		Semantic verbal fluency one category	22.00 (5.98)	24.12 (5.15)	
		Semantic verbal fluency category switch	13.94 (3.15)	14.79 (2.63)	
Beck Depression Inventory score			10.85 (9.80)	5.03 (3.94)	$t(42.1) = -3.166$, $p = 0.003$, $d = 0.779$
Interpersonal Reactivity Index		empathic concern	15.36 (2.43)	14.15 (2.41)	1) main effect of subscale $F(2.5,163.1) = 49.564$, $p < 0.001$, $\eta^2 = 0.436$
		personal distress	11.39 (3.59)	10.03 (2.39)	
		fantasy	12.33 (2.56)	12.00 (2.61)	
		perspective taking	14.30 (2.27)	14.91 (1.84)	
Multifaceted Empathy Test	Cognitive empathy	negative valence	10.00 (2.44)	11.61 (2.38)	1) main effect of group $F(1,64) = 7.830$, $p = 0.007$, $\eta^2 = 0.109$
		positive valence	11.67 (2.68)	12.97 (2.39)	2) main effect of valence $F(1,64) = 22.711$, $p < 0.001$, $\eta^2 = 0.262$
	Empathic concern	negative valence	129.06 (22.21)	117.85 (22.49)	1) main effect of group $F(1,64) = 5.043$, $p = 0.028$, $\eta^2 = 0.073$
		positive valence	118.58 (31.81)	101.79 (32.95)	2) main effect of valence $F(1,64) = 21.899$, $p < 0.001$, $\eta^2 = 0.255$
	Personal affective involvement	negative valence	121.24 (25.24)	108.76 (22.23)	1) main effect of group $F(1,64) = 4.183$, $p = 0.045$, $\eta^2 = 0.061$
		positive valence	112.27 (32.44)	98.21 (31.07)	2) main effect of valence $F(1,64) = 17.013$, $p < 0.001$, $\eta^2 = 0.210$
Social Problem Solving Task	Control questions (mean percent correct)		93.94 (12.73)	97.27 (5.17)	$t(42.3) = 1.393$, $p = 0.171$
	Detection of awkwardness (mean percent correct)		67.58 (28.62)	84.85 (15.03)	$t(48.4) = 3.070$, $p = 0.004$, $d = 0.756$
	Subjective degree of awkwardness (mean rating percent)		72.50 (19.45)	69.39 (11.05)	$t(50.7) = -0.798$, $p = 0.428$
	Fluency/number of solutions	SP	7.06 (2.81)	9.88 (3.37)	1) interaction of group and category $F(2.6,164.3) = 6.668$, $p = 0.001$, $\eta^2 = 0.094$
		S	4.48 (2.27)	4.79 (2.81)	SP solutions $t(64) = 3.692$, $p < 0.001$, $d = 0.909$
		P	3.97 (2.64)	3.79 (2.47)	
		N	1.39 (1.58)	1.42 (1.62)	2) main effect of category $F(2.6,164.3) = 116.814$, $p < 0.001$, $\eta^2 = 0.646$

Selection of optimal (SP) alternatives (mean percent correct)	50.30 (15.91)	61.21 (19.96)	$t(64) = 2.455$, $p = 0.017$, $d = 0.604$

Group differences were analyzed using *t*-tests and repeated-measures analyses of variances (ANOVAs) where appropriate. In the ANOVAs, group was considered as between-subject factor and fluency condition (German Regensburg verbal fluency test), subscale (Interpersonal Reactivity Index), valence (Multifaceted Empathy Test) or category (Social Problem Solving Task) as within-subject factor. Significant interactions were resolved by post-hoc *t*-tests to compare PCNSL patients and healthy controls with application of the Bonferroni-correction. Differences in the gender ratio were analyzed with the χ^2-test. SP: socially sensitive and practically effective, S: merely socially sensitive, P: merely practically effective, N: neither socially sensitive nor practically effective.

Results S3: Changes of the Result Pattern when Excluding PCNSL Patients Who Suffered from a Cerebral Tumor Relapse from the Analyses

Five out of 43 patients had suffered from a cerebral tumour relapse. Of these, two patients were treated at relapse with high-dose chemotherapy followed by autologous stem cell transplantation. Another three patients were treated with intensified conventional chemotherapy. Salvage treatment led to complete remission in all five patients at least one year before study participation. None of the five patients received whole brain radiotherapy at salvage. To rule out that sociocognitive impairment in the whole PCNSL group ($n = 43$) was caused by an interpretation bias since patients who suffered a cerebral tumor relapse received two lines of treatment, we repeated our analyses after excluding these five patients and their respective five matched healthy controls from the analyses ($n = 38$).

This changed the result pattern as follows: PCNSL patients and healthy controls (n = 38) differed in their estimated overall intelligence only at trend level ($p = 0.065$). In the repeated-measures ANOVA involving the four IRI subscales (within-subject factor) and group (between-subject factor) the interaction of subscale and group was still significant ($F(2.7,199.4) = 4.043$, $p = 0.010$, $\eta^2 = 0.052$). Post-hoc comparisons revealed that PCNSL patients now scored significantly lower on the perspective taking ($t(74) = 2.636$, $p = 0.010$, $d = 0.604$) subscale as a component of cognitive empathy as compared to healthy controls. This effect was only marginally significant ($p = 0.050$) in the group of 43 PCNSL patients. On the other hand, the significant difference ($n = 43$) on the personal distress subscale was only marginally significant ($p = 0.061$) in the group of 38 PCNSL patients. See Table S3 for the full report of the statistical analyses. The result pattern regarding all other assessments did not change when excluding patients having suffered from a cerebral relapse from the analyses.

Table S3. Demographic data, severity of depressive symptoms and performance concerning verbal fluency, self-reported and behavioral empathy and social problem solving of a reduced subgroup of patients with primary central nervous system lymphoma (PCNSL) and healthy controls. The table presents absolute values or mean scores with standard deviations presented in brackets as well as the test statistics.

		PCNSL Patients	Healthy Controls	Test Statistics
N		38	38	-
Median age at testing (years)		64.5 (range 37-82)	64.5 (range 35-80)	$t(74) = 0.031$, $p = 0.976$
Gender (female:male)		19:19	19:19	$\chi^2(1) = 0.0$, $p = 1.0$
Years of school		10.58 (2.25)	11.38 (1.85)	$t(71.3) = 1.698$, $p = 0.094$
Years of education		14.95 (3.70)	15.66 (3.38)	$t(74) = 0.873$, $p = 0.385$
Estimated overall intelligence		114.92 (13.37)	120.55 (12.52)	$t(72) = 1.873$, $p = 0.065$
German Regensburg verbal fluency test (number of words within one minute)	Phonematic verbal fluency	10.87 (3.87)	11.74 (4.01)	1) main effect of group $F(1,74) = 4.005$, $p = 0.049$, $\eta^2 = 0.051$ 1) main effect of condition $F(2,148) = 224.864$, $p < 0.001$, $\eta^2 = 0.752$
	Semantic verbal fluency one category	21.47 (5.83)	23.71 (5.39)	
	Semantic verbal fluency category switch	13.45 (3.30)	14.89 (2.66)	
Beck Depression Inventory score		10.24 (9.69)	5.16 (3.72)	$t(47.7) = - 3.017$, $p = 0.004$, $d = 0.692$
Interpersonal Reactivity Index	empathic concern	14.50 (2.68)	14.24 (2.58)	
	personal distress	11.00 (3.35)	9.74 (2.33)	

		fantasy	12.16 (2.81)	12.13 (2.83)	1) interaction of subscale and group $F(2.7,199.4) = 4.043$, $p = 0.010$, $\eta^2 = 0.052$
		perspective taking	13.79 (2.63)	15.21 (2.03)	perspective taking $t(74) = 2.636$, $p = 0.010$, $d = 0.604$ 2) main effect of subscale $F(2.7,199.4) = 51.341$, $p < 0.001$, $\eta^2 = 0.410$
Multifaceted Empathy Test	Cognitive empathy	negative valence	10.00 (2.52)	11.39 (2.41)	1) main effect of group $F(1,74) = 7.859$, $p = 0.006$, $\eta^2 = 0.096$
		positive valence	11.63 (2.77)	13.03 (2.38)	2) main effect of valence $F(1,74) = 30.574$, $p < 0.001$, $\eta^2 = 0.292$
	Empathic concern	negative valence	125.05 (24.15)	119.50 (22.24)	1) main effect of valence
		positive valence	112.92 (34.42)	106.42 (31.49)	$F(1,74) = 20.080$, $p < 0.001$, $\eta^2 = 0.213$
	Personal affective involvement	negative valence	115.13 (27.83)	110.13 (22.31)	1) main effect of valence
		positive valence	107.55 (33.38)	102.26 (29.67)	$F(1,74) = 10.015$, $p = 0.002$, $\eta^2 = 0.119$
Social Problem Solving Task	Control questions (mean percent correct)		95.26 (10.33)	96.84 (5.74)	$t(74) = 0.824$, $p = 0.413$
	Detection of awkwardness (mean percent correct)		68.68 (25.70)	82.63 (16.88)	$t(63.9) = 2.797$, $p = 0.007$, $d = 0.642$
	Subjective degree of awkwardness (mean rating percent)		72.96 (19.45)	72.92 (10.58)	$t(57.1) = -0.012$, $p = 0.991$
	Fluency/number of solutions	SP	7.03 (2.66)	9.71 (3.59)	1) interaction of group and category
		S	4.18 (2.22)	4.61 (2.65)	
		P	4.00 (2.42)	4.00 (2.51)	$F(2.5,188.6) = 6.420$, $p = 0.001$, $\eta^2 = 0.080$
		N	1.37 (1.51)	1.61 (1.78)	SP solutions $t(74) = 3.708$, $p < 0.001$, $d = 0.848$ 2) main effect of group $F(1,74) = 4.835$, $p = 0.031$, $\eta^2 = 0.061$ 3) main effect of category $F(2.5,188.6) = 134.254$, $p < 0.001$, $\eta^2 = 0.645$
	Selection of optimal (SP) alternatives (mean percent correct)		48.95 (19.00)	61.05 (19.14)	$t(74) = 2.767$, $p = 0.007$, $d = 0.635$

Group differences were analyzed using *t*-tests and repeated-measures analyses of variance (ANOVAs) where appropriate. In the ANOVAs, group was considered as between-subject factor and fluency condition (German Regensburg verbal fluency test), subscale (Interpersonal Reactivity Index), valence (Multifaceted Empathy Test) or category (Social Problem Solving Task) as within-subject factors. Significant interactions were analyzed using post-hoc *t*-tests to compare PCNSL patients and healthy controls with application of the Bonferroni-correction. Differences in gender ratio were analyzed with the χ^2-test. SP: socially sensitive and practically effective, S: merely socially sensitive, P: merely practically effective, N: neither socially sensitive nor practically effective.

Results S4: Differences of Sociocognitive Functions between Patients Who Had Undergone Resection and Those Who Did Not

Fifteen out of 43 patients had undergone resection of PCNSL ($n = 14$) or open biopsy ($n = 1$). Since the role of resection in PCNSL is debated and is possibly associated with neurological morbidity we additionally tested whether there were differences in sociocognitive functions between patients having undergone resection and those who did not. Since patient groups differed in sample sizes (15 versus 28 patients) we used non-parametric statistical methods i.e. Mann-Whitney-U-tests to compare patients who had undergone resection and those who did not.

When comparing PCNSL patients who had undergone resection or open biopsy with those who did not no significant differences (all *p*-values ≥ 0.251) occurred concerning sociocognitive performance (i.e. self-reported and behavioral empathy and social problem solving). See Table S4 for the full report of the statistical analyses.

Table S4. Severity of depressive symptoms and performance concerning self-reported and behavioral empathy and social problem solving of patients with primary central nervous system lymphoma (PCNSL) having undergone resection or

open biopsy and patients who did not. The table presents absolute values or mean scores with standard deviations presented in brackets as well as the test statistics.

			PCNSL Patients Having Undergone Resection	PCNSL Patients Not Having Undergone Resection	Test Statistics
N			15	28	-
Beck Depression Inventory score			11.33 (9.72)	9.75 (8.93)	*U* = 194.500, *Z* = - 0.396, *p* = 0.692
Interpersonal Reactivity Index		empathic concern	14.00 (3.05)	14.79 (2.60)	*U* = 173.000, *Z* = - 0.948, *p* = 0.343
		personal distress	11.13 (3.09)	11.29 (3.53)	*U* = 198.500, *Z* = - 0.295, *p* = 0.768
		fantasy	12.53 (2.67)	11.75 (3.00)	*U* = 189.500, *Z* = - 0.526, *p* = 0.599
		perspective taking	13.60 (3.04)	14.39 (2.41)	*U* = 186.500, *Z* = - 0.606, *p* = 0.545
Multifaceted Empathy Test	Cognitive empathy	negative valence	9.73 (2.63)	9.86 (2.42)	*U* = 190.500, *Z* = - 0.503, *p* = 0.615
		positive valence	10.87 (2.85)	11.21 (3.27)	*U* = 192.000, *Z* = - 0.462, *p* = 0.644
	Empathic concern	negative valence	126.20 (25.19)	124.11 (22.52)	*U* = 198.000, *Z* = - 0.306, *p* = 0.760
		positive valence	121.47 (32.95)	108.57 (32.33)	*U* = 165.000, *Z* = - 1.147, *p* = 0.251
	Personal affective involvement	negative valence	118.33 (30.07)	113.00 (26.39)	*U* = 174.500, *Z* = - 0.905, *p* = 0.365
		positive valence	115.20 (31.69)	103.71 (32.48)	*U* = 175.500, *Z* = - 0.879, *p* = 0.379
Social Problem Solving Task	Control questions (mean percent correct)		94.67 (13.02)	93.57 (11.29)	*U* = 195.500, *Z* = - 0.456, *p* = 0.648
	Detection of awkwardness (mean percent correct)		68.67 (25.88)	62.86 (28.66)	*U* = 187.000, *Z* = - 0.609, *p* = 0.543
	Subjective degree of awkwardness (mean rating percent)		76.91 (13.11)	70.57 (20.70)	*U* = 178.000, *Z* = - 0.816, *p* = 0.415
	Fluency/ number of solutions	SP	7.13 (2.20)	6.57 (2.95)	*U* = 177.500, *Z* = - 0.836, *p* = 0.403
		S	3.53 (2.26)	4.32 (2.21)	*U* = 166.000, *Z* = - 1.133, *p* = 0.257
		P	3.87 (2.00)	4.18 (2.87)	*U* = 207.000, *Z* = - 0.077, *p* = 0.939
		N	1.13 (1.13)	1.50 (1.58)	*U* = 189.000, *Z* = - 0.564, *p* = 0.573
	Selection of optimal (SP) alternatives (mean percent correct)		46.67 (22.25)	51.43 (19.95)	*U* = 186.500, *Z* = - 0.634, *p* = 0.526

Group differences were analyzed using Mann-Whitney-U-tests to compare sociocognitive performance of PCNSL patients who had undergone resection or open biopsy and those patients who did not. SP: socially sensitive and practically effective, S: merely socially sensitive, P: merely practically effective, N: neither socially sensitive nor practically effective

Results S5: Differences of Sociocognitive Functions between Patients Having Received High-Dose Chemotherapy Followed by Autologous Stem Cell Transplantation for Consolidation and Those Who Did Not

Seven out of 43 patients had received high-dose chemotherapy followed by autologous stem cell transplantation (HDASCT) for consolidation (i.e., in their first-line treatment). Since a recent study discussed an association between HDASCT for consolidation and delayed neurotoxicity in progression-free PCNSL patients we additionally tested whether there were differences in sociocognitive functions between patients having received HDASCT for consolidation and those who did not. Since patient groups differed in sample sizes (7 versus 36 patients), we used non-parametric statistical methods, i.e. Mann-Whitney-U-tests to compare PCNSL patients having received HDASCT for consolidation and those who did not.

When comparing PCNSL patients having received HDASCT for consolidation and those who did not only for the detection of awkwardness in social situations, significant group differences emerged ($p = 0.009$) with PCNSL patients having received HDASCT for consolidation performing better. Furthermore, patients having received HDASCT for consolidation rated the subjective degree of awkwardness of a social situation as significantly higher as compared to non-HDASCT-patients ($p = 0.005$). However, patients having received HDASCT for consolidation performed even better speaking against effects of delayed neurotoxicity. Concerning all other sociocognitive measures no significant group differences emerged (all p-values ≥ 0.078) between patients having received HDASCT for consolidation and those who did not. See Table S5 for the full report of the statistical analyses.

Table S5. Severity of depressive symptoms and performance concerning self-reported and behavioral empathy and social problem solving of patients with primary central nervous system lymphoma (PCNSL) having received high-dose chemotherapy followed by autologous stem cell transplantation for consolidation and those who did not. The table presents absolute values or mean scores with standard deviations presented in brackets as well as the test statistics.

			Patients Having Received HDASCT For Consolidation	Patients Without HDASCT For Consolidation	Test Statistics
N			7	36	-
Beck Depression Inventory score			13.14 (9.60)	9.75 (9.07)	U = 101.000, Z = - 0.824, p = 0.429
Interpersonal Reactivity Index		empathic concern	15.00 (2.83)	14.42 (2.77)	U = 113.500, Z = - 0.413, p = 0.687
		personal distress	10.43 (4.16)	11.39 (3.21)	U = 101.000, Z = - 0.827, p = 0.429
		fantasy	13.71 (2.06)	11.69 (2.93)	U = 72.500, Z = - 1.773, p = 0.078
		perspective taking	13.00 (3.06)	14.33 (2.54)	U = 91.000, Z = - 1.164, p = 0.263
Multifaceted Empathy Test	Cognitive empathy	negative valence	9.29 (1.80)	9.92 (2.58)	U = 116.500, Z = - 0.316, p = 0.760
		positive valence	12.00 (2.45)	10.92 (3.21)	U = 100.000, Z = - 0.861, p = 0.410
	Empathic concern	negative valence	124.71 (26.36)	124.86 (22.96)	U = 121.000, Z = - 0.165, p = 0.885
		positive valence	122.57 (49.11)	111.22 (29.19)	U = 91.000, Z = - 1.152, p = 0.263
	Personal affective involvement	negative valence	108.43 (32.04)	116.11 (26.85)	U = 105.500, Z = - 0.675, p = 0.508
		positive valence	117.14 (51.54)	105.89 (27.85)	U = 100.500, Z = - 0.839, p = 0.410
Social Problem Solving Task	Control questions (mean percent correct)		100.00 (0.00)	92.78 (12.56)	U = 80.500, Z = - 1.848, p = 0.137
	Detection of awkwardness (mean percent correct)		88.57 (10.69)	60.28 (27.51)	U = 48.000, Z = - 2.665, p = 0.009
	Subjective degree of awkwardness (mean rating percent)		88.14 (10.71)	69.79 (18.29)	U = 44.500, Z = - 2.683, p = 0.005
	Fluency/ number of solutions	SP	7.43 (3.64)	6.64 (2.52)	U = 110.500, Z = - 0.515, p = 0.617
		S	4.14 (3.13)	4.03 (2.08)	U = 123.000, Z = - 0.100, p = 0.936
		P	3.86 (3.02)	4.11 (2.53)	U = 118.500, Z = - 0.249, p = 0.809
		N	1.14 (0.90)	1.42 (1.52)	U = 119.500, Z = - 0.225, p = 0.834
	Selection of optimal (SP) alternatives (mean percent correct)		51.43 (25.45)	49.44 (19.99)	U = 117.500, Z = - 0.296, p = 0.784

Group differences were analyzed using Mann-Whitney-U-tests to compare sociocognitive performance of PCNSL patients having received high-dose chemotherapy followed by autologous stem cell transplantation for consolidation and patients who did not. SP: socially sensitive and practically effective, S: merely socially sensitive, P: merely practically effective, N: neither socially sensitive nor practically effective. HDASCT: high-dose chemotherapy followed by autologous stem cell transplantation

Results S6: Gender Differences in Sociocognitive Performance

We additionally analyzed if women and men performed differently on tasks assessing social cognition. Gender differences were analyzed using univariate and repeated-measures ANOVAs where appropriate. In the repeated-measures ANOVAs, fluency condition (German Regensburg verbal fluency test), subscale (IRI), valence (MET) or category (Social Problem Solving Task) respectively were considered as within-subject factors while gender (male versus female) and group (PCNSL patients versus healthy controls) were considered as between-subject factors. Age, years of school, years of education, estimated overall intelligence scores, BDI scores, performance on SCAMPS control questions, SCAMPS detection of awkwardness, SCAMPS subjective degree of awkwardness and performance on SCAMPS recognition of optimal solutions were considered as dependent variables in the univariate ANOVAs while group and gender were considered as independent variables. Results will be presented with a particular focus on gender differences (i.e. main effect of gender and interaction of gender and group).

The only analyses that yielded significant results for gender as a main effect or a significant interaction of group and gender were the analyses of SCAMPS detection of awkwardness and the subjective degree of awkwardness. In an univariate ANOVA involving SCAMPS detection of awkwardness as the dependent and group and gender as independent variables a significant main effect of gender occurred (F(1,82) = 7.983, p = 0.006, η^2 = 0.089) with females performing overall better (p = 0.006). Furthermore, there was a

significant interaction of group and gender (F(1,82) = 4.774, p = 0.032, η^2 = 0.055). In two separate t-tests holding group constant only in the group of PCNSL patients a significant difference between males and females occurred (t(39.8) = - 3.004, p = 0.005, d = 0.911) concerning detection of awkwardness. Male patients detected the awkward elements significantly less often accurately as compared to female PCNSL patients (p = 0.005). In the group of healthy controls, no such gender differences occurred (p = 0.560). In an univariate ANOVA involving SCAMPS subjective degree of awkwardness as the dependent variable and group and gender as independent variables a significant main effect of gender occurred (F(1,82) = 6.281, p = 0.014, η^2 = 0.071) with females presenting overall higher ratings (p = 0.014). Furthermore, there was a significant interaction of group and gender (F(1,82) = 3.948, p = 0.050, η^2 = 0.046). In two separate t-tests holding group constant only in the group of PCNSL patients a significant difference between males and females occurred (t(41) = −2.732, p = 0.009, d = 0.833). Male patients rated the degree of awkwardness significantly lower as compared to female patients (p = 0.009). In the group of healthy controls, no such gender differences were found (p = 0.651) concerning subjective degree of awkwardness. All other analyses did not yield any significant results (all p-values ≥ 0.120). See Tables S6 and S7 for descriptive data of males and females separately for PCNSL patients and healthy controls.

In conclusion, gender differences were present only for PCNSL patients concerning their ability to detect the awkward element in interpersonal situations (male < female) and for the rating of subjective awkwardness of such situations (male < female). However, for empathy and for the ability to freely produce and merely recognize appropriate solutions for difficult interpersonal situations (social problem solving) no gender differences were found.

Table S6. Demographic data, severity of depressive symptoms and performance concerning self-reported and behavioral empathy and social problem solving of female and male PCNSL patients. The table presents absolute values or mean scores with standard deviations presented in brackets.

			**Female PCNSL Patients**	**Male PCNSL Patients**
N			21	22
Median age at testing (years)			63 (range 37-82)	66 (range 44-83)
Years of school			10.95 (2.33)	10.18 (2.06)
Years of education			14.67 (3.04)	15.16 (4.29)
Estimated overall intelligence			113.20 (14.64)	114.05 (13.35)
German Regensburg verbal fluency test (number of words within one minute)		Phonematic verbal fluency	12.14 (4.04)	9.64 (3.75)
		Semantic verbal fluency one category	21.76 (5.74)	20.00 (5.84)
		Semantic verbal fluency category switch	13.29 (2.26)	13.45 (4.08)
Beck Depression Inventory score			12.10 (10.91)	8.59 (6.86)
Interpersonal Reactivity Index		empathic concern	14.38 (2.73)	14.64 (2.84)
		personal distress	12.24 (3.19)	10.27 (3.27)
		fantasy	12.57 (2.56)	11.50 (3.13)
		perspective taking	14.29 (3.16)	13.95 (2.08)
Multifaceted Empathy Test	Cognitive empathy	negative valence	10.10 (2.70)	9.55 (2.24)
		positive valence	11.24 (2.97)	10.95 (3.29)
	Empathic concern	negative valence	128.95 (23.63)	120.91 (22.63)
		positive valence	115.76 (34.84)	110.50 (31.23)
	Personal affective involvement	negative valence	117.76 (27.44)	112.09 (27.89)
		positive valence	108.33 (32.37)	107.14 (32.99)
Social Problem Solving Task	Control questions (mean percent correct)		94.29 (13.63)	93.64 (10.02)
	Detection of awkwardness (mean percent correct)		76.67 (22.21)	53.64 (27.87)
	Subjective degree of awkwardness (mean rating percent)		80.12 (11.56)	65.77 (21.25)
	Fluency/number of solutions	SP	7.43 (2.66)	6.14 (2.64)
		S	4.33 (2.22)	3.77 (2.27)
		P	4.48 (2.64)	3.68 (2.51)
		N	1.19 (1.47)	1.55 (1.41)

Selection of optimal (SP) alternatives (mean percent correct)		50.48 (22.47)	49.09 (19.25)

SP: socially sensitive and practically effective, S: merely socially sensitive, P: merely practically effective, N: neither socially sensitive nor practically effective.

Table S7. Demographic data, severity of depressive symptoms and performance concerning self-reported and behavioral empathy and social problem solving of female and male healthy participants. The table presents absolute values or mean scores with standard deviations presented in brackets.

			**Female Healthy Participants**	**Male Healthy Participants**
N			21	22
Median age at testing (years)			63 (range 35-80)	66.5 (range 47-80)
Years of school			11.64 (1.71)	11.14 (1.98)
Years of education			15.62 (3.67)	15.64 (2.99)
Estimated overall intelligence			118.71 (12.67)	124.50 (12.30)
German Regensburg verbal fluency test (number of words within one minute)		Phonematic verbal fluency	12.10 (3.67)	11.32 (4.73)
		Semantic verbal fluency one category	23.71 (6.08)	23.41 (4.25)
		Semantic verbal fluency category switch	15.43 (2.77)	14.73 (2.71)
Beck Depression Inventory score			5.43 (4.46)	4.91 (2.91)
Interpersonal Reactivity Index		empathic concern	14.67 (2.44)	13.95 (2.54)
		personal distress	10.10 (2.34)	9.50 (2.44)
		fantasy	12.62 (2.40)	12.00 (3.07)
		perspective taking	14.81 (2.11)	15.41 (1.84)
Multifaceted Empathy Test	Cognitive empathy	negative valence	11.90 (2.41)	11.05 (2.30)
		positive valence	13.81 (2.18)	12.50 (2.24)
	Empathic concern	negative valence	120.19 (22.07)	119.50 (21.48)
		positive valence	109.05 (31.23)	101.68 (31.40)
	Personal affective involvement	negative valence	113.33 (22.39)	107.64 (21.26)
		positive valence	104.62 (30.33)	99.14 (28.97)
Social Problem Solving Task	Control questions (mean percent correct)		97.38 (5.39)	96.82 (5.68)
	Detection of awkwardness (mean percent correct)		84.76 (14.01)	81.82 (18.42)
	Subjective degree of awkwardness (mean rating percent)		73.48 (12.73)	71.82 (11.10)
	Fluency/number of solutions	SP	10.29 (3.58)	9.36 (3.30)
		S	5.48 (2.68)	4.27 (2.47)
		P	4.00 (2.14)	4.23 (2.86)
		N	1.19 (1.25)	1.91 (2.09)
	Selection of optimal (SP) alternatives (mean percent correct)		60.95 (20.47)	61.82 (19.43)

SP: socially sensitive and practically effective, S: merely socially sensitive, P: merely practically effective, N: neither socially sensitive nor practically effective.

Results S7: Additional Non-Parametric Analyses

To assess whether the effects were robust, non-parametric analyses for all sociocognitive measures were additionally computed. Mann-Whitney U-tests were calculated to compare PCNSL patients and healthy controls with regard to the subscales of the IRI, the MET cognitive empathy scores, the MET empathic concern scores, the MET personal affective involvement scores (all MET scores separately for both valences), the Social Problem Solving Fluency Task control questions, detection of awkwardness, subjective degree of awkwardness, solution fluency and recognition of optimal solutions. Only for the IRI subscale perspective taking the previously marginally significant group difference was abolished when using non-parametric tests ($p = 0.126$). However, it has to be kept in mind that this group difference also did not withstand the applied Bonferroni-correction previously. The result pattern for all other sociocognitive measures was comparable when using non-parametric or parametric statistical methods (Table S8). Therefore, we assume that the effects on sociocognitive performance were robust.

Table S8. Non-parametric statistics concerning self-reported and behavioral empathy and social problem solving of patients with primary central nervous system lymphoma (PCNSL) and healthy controls.

			**Test Statistics**
Interpersonal Reactivity Index		empathic concern	$U = 888.500$, $Z = -0.313$, $p = 0.754$
		personal distress	$U = 665.500$, $Z = -2.251$, $p = 0.024$
		fantasy	$U = 886.000$, $Z = -0.335$, $p = 0.738$
		perspective taking	$U = 749.000$, $Z = -1.531$, $p = 0.126$
Multifaceted Empathy Test	Cognitive empathy	negative valence	$U = 558.000$, $Z = -3.191$, $p = 0.001$
		positive valence	$U = 572.500$, $Z = -3.064$, $p = 0.002$
	Empathic concern	negative valence	$U = 807.500$, $Z = -1.011$, $p = 0.312$
		positive valence	$U = 810.000$, $Z = -0.989$, $p = 0.323$
	Personal affective involvement	negative valence	$U = 788.000$, $Z = -1.179$, $p = 0.238$
		positive valence	$U = 840.500$, $Z = -0.726$, $p = 0.468$
Social Problem Solving Task	Control questions (mean percent correct)		$U = 854.000$, $Z = -0.773$, $p = 0.439$
	Detection of awkwardness (mean percent correct)		$U = 567.000$, $Z = -3.258$, $p = 0.001$
	Subjective degree of awkwardness (mean rating percent)		$U = 847.500$, $Z = -0.666$, $p = 0.506$
	Fluency/ number of solutions	SP	$U = 454.000$, $Z = -4.084$, $p < 0.000$
		S	$U = 768.500$, $Z = -1.359$, $p = 0.174$
		P	$U = 906.000$, $Z = -0.161$, $p = 0.872$
		N	$U = 910.500$, $Z = -0.126$, $p = 0.900$
	Selection of optimal (SP) alternatives (mean percent correct)		$U = 650.500$, $Z = -2.487$, $p = 0.013$

Group differences were analyzed using Mann-Whitney-U-tests to compare sociocognitive performance of PCNSL patients and healthy controls. SP: socially sensitive and practically effective, S: merely socially sensitive, P: merely practically effective, N: neither socially sensitive nor practically effective.
